# Letrozole supplementation during controlled ovarian stimulation in expected high responders: a pilot randomized controlled study

**DOI:** 10.1186/s12958-019-0483-x

**Published:** 2019-05-10

**Authors:** Xiaoyi Yang, Ge Lin, Guangxiu Lu, Fei Gong

**Affiliations:** 1 Institute of Reproducitve and Stem Cell Engineering, Basic Medicine College, Central South University, Changsha, 410078 China; 2 Reproductive and Genetic Hospital of CITIC-XIANGYA, Changsha, China; 3 Key Laboratory of Reproductive and Stem Cell Engineering, National Health and Family Planning Commission, Changsha, China

**Keywords:** Letrozole, Progesterone, Randomized study, High responders, In vitro fertilization

## Abstract

**Background:**

Almost all of the previous studies related with co-administration of letrozole in IVF cycles were performed in poor responders and letrozole may reduce the total gonadotropin dose required for ovarian stimulation, and the pregnancy rate did not decrease in poor responders. This study aimed to assess whether high responders co-treatment with letrozole reduced supraphysiological late follicular phase estradiol levels and the incidence of premature progesterone elevated at the end of the follicular phase, thereby impacting positively on endometrial receptivity.

**Methods:**

A randomized parallel controlled study in a university-affiliated center include 130 high responders between October 2015 and August 2016. The patients were randomized on the first stimulation day of the IVF cycle and from stimulation day 5 receive letrozole (group A) or without letrozole treatment (group B).

**Results:**

Although estradiol levels were significantly lower in the letrozole group (group A) (*P* < 0.001), progesterone elevation (> 1.5 ng/mL was considered as a rise) on the day of hCG triggering (15.4, 7.7%) was not statistically significant (*P* = 0.170). RecFSH, the recovery rate of eggs, the high-quality embryo rate, and the thickness of endometrium (*P* = 0.776) were similar between the letrozole group(group A) and control groups (group B). Clinical pregnancy rates were 53.1% (26/49) and 72.9% (35/48) in the letrozole and control groups, respectively, with a statistical significance (*P* = 0.043).Live birth rates were 42.9% (21/49) and 62.5% (30/48),showed a marginally significant difference (*P* = 0.053). The miscarriage rate did not significantly differ between the two groups.

**Conclusions:**

In this pilot study, letrozole supplementation could not reduce the incidence of premature progesterone rise during the late follicular phase in stimulated in vitro fertilization cycles in expected high responders, producing a harmful effect on the pregnancy outcome.

**Trial registration:**

China Clinical Trial Registration Center: ChiCTR-IPR-15006211 URL of the trial registry record: http://www.chictr.org.cn/showproj.aspx?proj=10731. Trial registration date: 8 April, 2015. Date of first patient’s enrolment: 5 October, 2015.

## Background

The effects of gonadotropin ovarian stimulation is a core component of in vitro fertilization (IVF) treatment. It was demonstrated that this might in part reflect the detrimental effects of gonadotropin ovarian stimulation on oocyte, embryo, and endometrial quality [1–3]. The impact of supraphysiological progesterone levels on endometrial maturation and receptivity is still a major concern in assisted reproduction. Increased serum progesterone levels in the late follicular phase are associated with reduced implantation rate [4]. Whether it is gonadotropin-releasing hormone (GnRH) agonist or GnRH antagonist used for pituitary downregulation, the progesterone levels of more than 1.5 ng/mL on the day of human chorionic gonadotropin (hCG) administration in IVF-stimulated cycles were associated with lower pregnancy rates [5–7].

High estradiol (E2) levels have been implicated as another causative factor. Recent studies have shown that patients with high E2 levels in the late follicular phase have significant progesterone elevation [8, 9]. Analysis of more than 60,000 cycles showed that serum E2 levels and the number of mature oocytes collected on the day of hCG administration also showed a statistical relationship with elevated progesterone and was associated with a lower pregnancy rate [10].

This mechanism might be related to the high reactivity of the ovary in the IVF stimulation cycle. An increased number of mature follicles and granulosa cells are present in high responders. It is suggested that a mechanism might exist to increase the progesterone levels associated with high ovarian responses during controlled ovarian stimulation. This idea was confirmed [11, 12], indicating that the incidence of progesterone elevation on the day of hCG administration was related to the ovarian response.

Milder stimulation regimens have been advocated as a means of mitigating these effects [13], but a recent study showed that the E2 levels were less affected by late-start mild protocols [14]. The question, therefore, remains whether medications of ovarian stimulation can ameliorate the negative effects on endometrial receptivity. It was proposed that this might be achieved using aromatase inhibitors (AIs), which inhibit the intraovarian aromatization of androgens to estrogens. Combining less gonadotropin stimulation to produce adequate numbers of oocytes with a simple oral adjuvant therapy aimed at preventing excessive E2 and progesterone levels, the resultant stimulation protocol might provide the “best of both worlds” solution, in which adequate numbers of oocytes were obtained, but not at the cost of detrimental impacts on oocytes, embryos, or endometrial quality. AIs have an established role in preventing the recurrence of estrogen-dependent tumors such as breast cancer [15]. The safety of long-term AIs has been repeatedly demonstrated in women conceiving after years of AI treatment [16].

It was widely used for poor responders because of lower total dosage and shorter duration [17, 18]. However, it was not routinely used for high responders in IVF stimulation cycles. So far, few studies have investigated its effect on the progesterone levels.

This study aimed to assess whether co-treatment with letrozole reduced supraphysiological late follicular phase E2 levels and the incidence of premature progesterone elevated at the end of the follicular phase, thereby impacting positively on endometrial receptivity.

## Methods

### Patients

This was a single-center randomized parallel controlled study in university-affiliated tertiary hospital reproductive center in Changsha, China. In this study, principles outlined inthe Declaration of Helsinki were followed and informed consent of all patientswas obtained.

The recruited participants were randomly assigned to two groups in a 1:1 ratio.

The Institutional Review Boards at Ethics Committee of Reproductive and Genetic Hospital CITIC-Xiangya approved the agreement. All participants signed the written informed consent. They participated in one study only and did not receive any funding for their participation in the study.

### Participants

Altogether, 130 women undergoing IVF–embryo transfer (ET) treatment were enrolled. High responders were defined as those with an antral follicle count (AFC) of at least 15.The inclusion criteria were as follows: (1) medical indication for IVF treatment; (2) AFC between 15 and 23; (3) age 21–35 years; (4) at the first or the second treatment cycle; and (5) the body mass index (BMI) 18–28. The exclusion criteria were as follows: (1) medical contraindication to IVF treatment (< 4 oocytes obtained); (2) previous documented poor response to ovary in IVF-stimulated cycles; (3) presence of endometriosis disease or uterine malformations; and (4) a history of unexplained miscarriages.

### Randomization

On the first day of the IVF cycle, eligible patients were recruited and subjected to ultrasound scans. They were then randomly divided into two groups based on the sealed computer-generated list: letrozole and control groups. Random number list was generated by research department staff of the hospital and provided to the study nurse in a sealed envelope. After the patients were recruited, the assignment order was hidden until the research nurse specified the intervention. Physicians and embryologists were blinded to the assignments.

### Treatment

All participants were subcutaneously administered 0.05 mg diphereline acetate (Diphereline 0.1 mg, Ipsen) daily from midluteal phase of the preceding IVF-ET cycle to hCG administration day with a standard short-acting GnRH-agonist protocol, and the initial dose of gonadotropin was determined based on the characteristics of each patient (age, BMI, and AFC).

In the letrozole group(group A, *n* = 65), the participants received the standard GnRH-agonist protocol, which was daily intramuscular injections of 100–225 IU recFSH from stimulation day 1 (gonafal; Merck, Germany) until the day of hCG administration and co-treatment with letrozole 2.5 mg (Jiangsu, Hengrui, China) daily from stimulation day 5 until the day of hCG administration. In the control group (group B, *n* = 65), 100–225 IU of recFSH was injected intramuscularly daily from day 1 of stimulation (gonafal; Merck, Germany) to the day of hCG administration. A standard luteal phase support with progesterone was provided. Crinone 8% (Progesterone Vaginal Gel) was initiated following retrieval by all 155 patients.

From the first day of the IVF cycle, ovarian responses were monitored by continuous vaginal scans and hormone measurements. hCG at a dose of 6500 IU (Merck) was given when at least three follicles were 18–20 mm in diameter. Oocyte retrieval was 35–36 h after the hCG injection.

### Outcome

The primary end point of the study was as follows: The percentage of patients had progesterone elevation on the day of hCG triggering (≥1.5 ng/mL was defined as elevation).

The secondary outcomes were as follows: (1) Serum E2 and progesterone levels on the hCG administration day (or the day before) and day 6 after ET; (2) Number of follicles > 12 mm on the day of hCG administration (or the day before) and oocytes obtained; and (3) Total IU of FSH used per cycle. (4) Clinical pregnancy rate.

Ultrasound-guided oocytes retrieved were performed with a 6.5-MHz vaginal probe and a 16-gauge single-channel needle (Cook IVF, Cook, Australia). The recovered oocytes were inseminated according to the conventional IVF/intracytoplasmic sperm injection. After 16–18 h, they were checked for fertilization. When two pronuclei were visible, normal fertilization was considered. One or two normal embryos were transferred into the uterine cavity 3 days after removal. Blood was collected within 6 days after hCG administration to measure serum E2 and progesterone concentrations. The serum E2 and progesterone concentrations were rechecked 11 days after hCG administration.

Tables 1 and 2 summarize the baseline characteristics of the patients, clinical parameters from ovarian stimulation to embryo transfer, serum progesterone levels and clinical pregnancy rate.

Serum pregnancy test was performed 19 days after hCG administration. Pregnancies were diagnosed by an increasing concentration more than 200 IU/mL of serum β-hCG 14 days after ET. Clinical pregnancies were defined as ultrasound imaging of one or more gestational sacs or clear clinical signs of pregnancy.

First-trimester miscarriage was defined as a loss of clinical pregnancy before 70 days after ET. Abortion during late pregnancy was defined as the loss of clinical pregnancy after 70 days after ET. Live birth was defined as the birth of one or two living healthy children.

### Statistical analysis

It was hard to define sample size based on all of the previous studies, because the data about AIs in high responders were quite limited in assisted reproductive technology cycles. Some studies found a difference of elevated *P* > 1.5 ng/mL increased with the ovarian response with 1–5, 6–9, 10–13, 14–18, and > 18 oocytes (4.5, 3.9, 8.3, 12.1, and 19.0%). Based on these reports, we supposed that the incidence of high serum progesterone levels on the day of hCG administration from 15 to 2% [11, 19], with 80% power at an *α* of 0.05, 57 patients were required per arm. Taking into account a drop-out rate of 15%, 65 patients were required.

Quantitative indicators subject to normal distribution were presented as mean ± standard deviation. The *P* values were calculated using independent-sample *t* tests. Median (Min, Max) was not used to describe the normal distribution, and *P* value was calculated using the nonparametric test. The categorical indicators were described using frequency, and two independent samples were compared using the chi-square test or Fisher’s exact test.Social Sciences version 22.0 was used. A *P* value of < 0.05 was considered statistically significant.

## Results

This study was approved by the Ethics Committee of Reproductive and Genetic Hospital CITIC-Xiangya on November 20, 2014 (LL-SC-SG-2014-014) and registered in Changsha (ChiCTR-IPR-15006211) on April 8, 2015.The study was started on October 2015. The first subject was enrolled on October 5, 2015, and the last one on August 9, 2016.

**Fig. 1:**
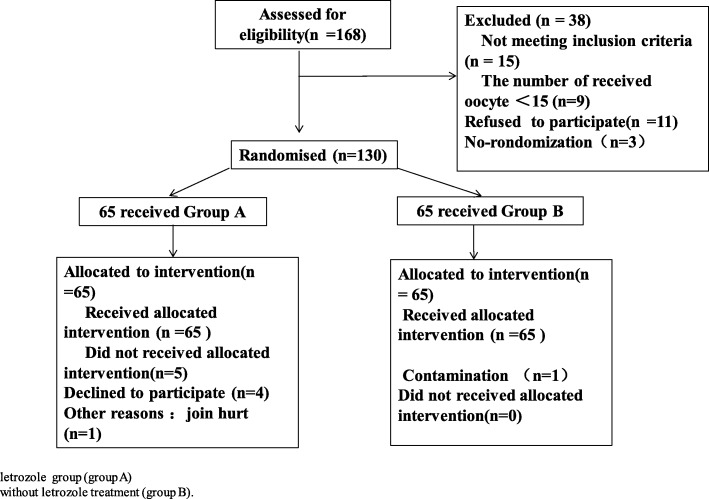
Flow chart on subject disposition

The flowchart of this trail is shown in Fig. 1.

The number of patients who withdrew from the study was 5 out of 65 (7.7%) in the Group A and 1 out of 65 (1.5%) in the Group B (Fig. 1).

Reasons for withdrawal were as follows: refusing to participate (*n* = 4) and joint pain (*n* = 1) in Group A (the study group) and contamination (*n* = 1) in Group B (the control group).

No significant differences were found in the age of the women, duration of infertility, BMI, types of infertility, AFC, or baseline hormonal profile (FSH) between the two groups (Table 1).

**Table 1:** Baseline characteristics of patients

	Letrozole	No letrozole	Wilcoxon *W*/*t*/*X*^*2*^	*P*
*N*	65	65		
Age of women (year)	28 (21,35)	29 (23,36)	−1.182	0.237
Body mass index (kg/m^2^)	21.76 ± 2.4	22.13 ± 2.45	− 0.878	0.382
Duration of infertility (year)	3 (1,10)	3 (1,12)	−0.262	0.793
Number of embryo transfer	2 (0,2)	2 (0,2)	−0.229	0.819
Infertility type			0.281	0.596
Primary infertility	30 (46.15%)	27 (41.54%)		
Secondary infertility	35 (53.85%)	38 (58.46%)		
Antrol follicle count	22 (14,23)	22 (12,30)	−0.592	0.554
Mean baseline hormone values (Day3) FSH (UI/L)	5.36 (2.72,11.27)	5.51 (3.24,12.01)	−0.17	0.865
Progesterone (nmol/L)	0.27 (0.1,0.95)	0.27 (0.1,1.07)	−0.142	0.887

### Primary outcomes

The patients had progesterone elevation (a level more than 1.5 ng/mL was considered as PE) on the day of hCG triggering (15.4, 7.7%), which was not statistically significant (*P* = 0.170).

### Secondary outcomes

Progesterone levels were 1.03 (0.8, 1.26) and 0.91 (0.71, 1.15) ng/mL (*P* < 0.001) on the day of hCG administration, while serum E2 levels in letrozole group were significantly lower than those in the control group; progesterone/E2 (*P* < 0.001) increased. Serum E2 and progesterone levels on the day before hCG administration and on day 6 after ET were similar between the letrozole and control groups.

RecFSH, the recovery rate of eggs, the fertilization rate, the high-quality embryo rate, and the thickness of endometrium were similar between the letrozole and control groups. No difference was found in the total IU of FSH used per cycle (*P* = 0.482).

Clinical pregnancy rates were 53.1% (26/49) and 72.9% (35/48) in the letrozole and control groups, respectively, which were statistically significant (*P* = 0.043). Live birth rates were 42.9% (21/49) and 62.5% (30/48) in the letrozole and control groups. (*P* = 0.053). Live birth rates were 42.9% (21/49) and 62.5% (30/48), showed a marginally significant difference (*P* = 0.053).The miscarriage rate did not significantly differ between the two groups (Table 2).

**Table 2:** Clinical/oocyte/embryo parameters from ovarian stimulation to embryo transfer relationship between serum progesterone levels and clinical pregnancy rates

	Group A	Group B	Wilcoxon *W*/*t*/*X*^*2*^	*P*
Patients with P elevation on day of administraition day n(%)	15.4% (10/65)	7.7% (5/65)	1.884	0.170
RecFSH	1275 (0,2700)	1350 (787.52175)	−0.703	0.482
RecFSH/day	137.5 (0,225)	142.5 (98.44,197.73)	−1.735	0.083
Recovery rate of eggs	13 (1,28)	12 (3,34)	−1.516	0.129
Duration of GN(day)	9 (6,19)	9 (7,13)	−1.293	0.196
Fertilized egg number	8.72 ± 4.26	8.63 ± 4.15	0.132	0.895
Fertilization rate	69.2% (0,100%)	78.5% (0,100%)	−1.493	0.135
Number of cleavage	8.57 ± 4.23	8.53 ± 4.19	0.051	0.959
Cleavage rate	100% (100,100%)	100% (100,100%)		
High-quality embryo	5 (0,13)	6 (0,14)	−0.879	0.379
Rate of production of high-quality embryos	60% (0,100%)	70% (0,100%)	−1.21	0.226
P on the day of hCG administration (ng/mL)	1.05 (0.3,2.61)	0.95 (0.15,2.06)	−2.047	0.041
E2 on the day of hCG administration (pg/mL)	1404.28 (431.36227)	2867 (1043.62,7198)	−7.131	< 0.001
P/E2 on the day of hCG administration	0.73 (0.066,1.86)	0.317 (0.043,1.84)	−6.931	< 0.001
E2 on 1 day before the day of hCG administration	546.95 (0,2611)	1102 (0,5488)	−2.055	0.040
P on 1 day before the day of hCG administration	0.545 (0,1.65)	0.52 (0,1.83)	−0.33	0.741
P on 6th day post-ET (ng/mL)	9.025 (0,96.3)	9.08 (0,44.86)	−0.407	0.984
E2 on 6th day post-ET (ng/mL)	103.4 (0,4737)	127.75 (0,2656)	−0.158	0.875
Thickness of endometrium	12.1 (9,17)	12.2 (8.3,17.8)	−0.284	0.776
Clinical pregnancy rate	53.1% (26/49)	72.9% (35/48)	4.096	0.043
Implantation rate	35.1% (33/94)	47.4% (45/95)	2.931	0.087
Incidence of OHSS	0% (0/65)	1.5% (1/65)	1.008	1.000
Live birth rate	42.9% (21/49)	62.5% (30/48)	3.752	0.053
First trimester miscarriage	7.7% (2/26)	5.7% (2/35)	0.095	1.000
Second trimester miscarriage	7.7% (2/26)	5.7% (2/35)	0.095	1.000

Note: quantitative indicators subject to normal distribution were presented as mean ± standard deviation. Median (Min, Max) was not used to describe the normal distribution. Group A: letrozole 2.5 mg/day from cycle day 5 to the day before hCG administration. Group B: no letrozole; *ET* Embryo transfer, *RecFSH* Recombinant FSH, *OHSS* Ovarian hyperstimulation syndrome

## Discussion

This study revealed that letrozole supplementation could not reduce the incidence of premature progesterone rise/elevation (*P* > 1.5 ng/mL) in the late follicular phase in stimulated IVF cycles in high responders.

Although progesterone had a key role in the IVF stimulation cycle and was particularly important for endometrial receptivity, the harmful effects of its serum level in the late follicular phase have been controversial and a cause of concern [20–22].

The effect of letrozole has been controversial in stimulated IVF cycles. Especially for poor responders, although the pregnancy rate did not increase significantly, the cancellation rate [23] and the miscarriage rate [16]decreased in the IVF-stimulated cycle with letrozole. When given to infertile women, decreased levels of estrogen led to increased FSH secretion from the pituitary gland. This increased FSH stimulated follicular development and was the basis of AI for ovulation induction [24, 25]. It is generally recommended to give letrozole for 5 days for ovulation induction or superovulation because its half-life is 45–72 h. The main side effects include a mild headache and muscle or joint pain [26, 27]. However, in a late large RCT study in N Engl J Med shows, ovarian stimulation with letrozole in women with unexplained infertility resulted in a lower frequency of live birth [28].

In the present study, no difference was found in the incidence of progesterone elevation in the late follicular phase in patients treated with the GnRH-agonist protocol. Previous randomized trials of GnRH agonists co-treated with letrozole did not measure the late follicular progesterone levels.

Almost all of the previous studies related with co-administration of letrozole in IVF cycles were performed in poor responders [29, 30]. The data about AIs in high responders were quite limited in assisted reproductive technology cycles. In a pilot study, letrozole stimulation from endometrial preparations in PCOS patients in frozen embryo transfer cycles may have better results than hormone manipulation or hMG stimulation. [28]. To date, no studies have reported the use of letrozole to specifically improve clinical outcome for high responders in IVF stimulation cycles(for example: progesterone levels). In this study, in addition to lower serum E2 levels, a little difference was found in the levels of other hormones that responded to high-response co-treatment with letrozole compared with the patients who did not take it.

Progesterone in the early follicular phase has an adrenal origin [31, 32]. In a recent study, decreasing progesterone levels in early follicular phase seems to be beneficial for cumulative live birth rates. However, in the late follicular phase, progesterone is mainly derived from mature follicles. Higher daily FSH dose was the factor most related to the occurrence of serum progesterone elevation [6, 10, 33]. The meta-analysis of Papanikolao [5, 32] showed that AI administration induced an acute hypoestrogenic state that released the hypothalamic–pituitary axis from estrogenic negative feedback, in turn increasing FSH secretion and ovarian follicle development [27]. Letrozole appeared to have the potential to reduce the total gonadotropin dose required for ovarian stimulation. In this study, recFSH was similar between the letrozole and control groups. The patients had progesterone elevation on the day of hCG administration, which was not statistically significant. More eggs were recovered in the letrozole groups. Considering the fact that high progesterone levels were positively related to the number of mature follicles, it was possible that low responders with a lower expression of FSH receptor in the granulosa cells were demonstrated [33]. Contrary to this situation, high responders showed the overexpression of FSH receptors in their follicular granulosa cell population [34, 35] and might be the reason why letrozole could not reduce the total gonadotropin dose needed for ovarian stimulation in hyper-responders.

Two successive enzymatic reactions convert progesterone into androgen (by 17αhydroxylase and C17, 20 lyase) and then again into estrogen by aromatase [36, 37]. In our study, add to letrozole means aromatase enzyme is blocked, and it may lead to accumulation of such as progesterone, testosterone, and 17α-progesterone on progesterone levels.

This study suggested that co-treatment with letrozole in high responders for agonist protocols could not reduce the incidence of premature progesterone increase in the late follicular phase in stimulated IVF cycles, producing a harmful effect on the pregnancy outcome. Therefore, this study did not continue to expand.

This study found that even higher levels of progesterone in high responders might have a negative impact on the implantation rate of high-quality cleavage-stage embryos [38]. Therefore, the question was what should be done in patients with advanced ovarian progesterone levels. An alternative means of reducing the exposure of gametes and endometrium to supraphysiological levels of progesterone was to selectively freeze, thaw, and transfer all embryos into a nonstimulated cycle. This option of “compartmentalizing” IVF treatment attracted increasing attention as recently published data suggested that clinical outcomes after embryo vitrification were satisfactory. However, in China, most patients prefer to choose fresh-cycle transplantation instead of cryopreservation for economic reasons and psychological factors.The question, therefore, remains whether medications for ovarian stimulation can ameliorate the negative effects on endometrial receptivity. The limitations of this pilot study were the use of high responders in GnRH-agonist cycles and the small sample size. The definition of high responder is just based on antral follicle counts at baseline. A further high-quality study is required to validate the findings. Moreover, the administration of letrozole offered a new insight to high-responders.

## Conclusions

Therefore, the fact that co-treatment with letrozole in high responders cannot be a positive factor for the IVF outcome must be considered seriously.The effect of elevated progesterone levels in high responders on clinical outcomes might be due to a balance between the two antagonistic factors: the possibility of obtaining more eggs and ovarian response and the endometrial preterm birth-impaired possibility to progesterone.

## Abbreviations

AFC: qAntral follicle count
AIs: Aromatase inhibitors
BMI: body mass index
E2: Estradiol
ET: Embryo transfer
FET: Frozen embryo transfer
FSH: Follicle stimulating hormone
GnRH: Gonadotropingonadotropin-releasing hormone
HCG: Human chorionic
IVF: In vitro fertilization
OHSS: Ovarian hyperstimulation
P: progesterone
